# Chemical immobilization of adult female Weddell seals with tiletamine and zolazepam: effects of age, condition and stage of lactation

**DOI:** 10.1186/1746-6148-2-8

**Published:** 2006-02-09

**Authors:** Kathryn E Wheatley, Corey JA Bradshaw, Robert G Harcourt, Lloyd S Davis, Mark A Hindell

**Affiliations:** 1 Antarctic Wildlife Research Unit, School of Zoology, University of Tasmania, Private Bag 05, Hobart, Tasmania 7001, Australia; 2 School for Environmental Research, Institute of Advanced Studies, Charles Darwin University, Darwin, Northern Territory 0909, Australia; 3 Marine Mammal Research Group, Graduate School of the Environment, Macquarie University, Sydney, New South Wales 2109, Australia; 4 Department of Zoology, University of Otago, P.O. Box 56, Dunedin, New Zealand

## Abstract

**Background:**

Chemical immobilization of Weddell seals (*Leptonychotes weddellii*) has previously been, for the most part, problematic and this has been mainly attributed to the type of immobilizing agent used. In addition to individual sensitivity, physiological status may play an important role. We investigated the use of the intravenous administration of a 1:1 mixture of tiletamine and zolazepam (Telazol^®^) to immobilize adult females at different points during a physiologically demanding 5–6 week lactation period. We also compared performance between IV and IM injection of the same mixture.

**Results:**

The tiletamine:zolazepam mixture administered intravenously was an effective method for immobilization with no fatalities or pronounced apnoeas in 106 procedures; however, there was a 25 % (one animal in four) mortality rate with intramuscular administration. Induction time was slightly longer for females at the end of lactation (54.9 ± 2.3 seconds) than at post-parturition (48.2 ± 2.9 seconds). In addition, the number of previous captures had a positive effect on induction time. There was no evidence for effects due to age, condition (total body lipid), stage of lactation or number of captures on recovery time.

**Conclusion:**

We suggest that intravenous administration of tiletamine and zolazepam is an effective and safe immobilizing agent for female Weddell seals. Although individual traits could not explain variation in recovery time, we suggest careful monitoring of recovery times during longitudinal studies (> 2 captures). We show that physiological pressures do not substantially affect response to chemical immobilization with this mixture; however, consideration must be taken for differences that may exist for immobilization of adult males and juveniles. Nevertheless, we recommend a mass-specific dose of 0.50 – 0.65 mg/kg for future procedures with adult female Weddell seals and a starting dose of 0.50 mg/kg for other age classes and other phocid seals.

## Background

Immobilization of captive and free-ranging pinnipeds is often required for biological studies, translocation or the examination of sick or injured animals. However, pinnipeds present unique problems when using chemical immobilization agents because they have evolved specific adaptations in their respiratory, cardiovascular and thermoregulatory systems enabling them to dive for extended periods. These adaptations can exacerbate problems associated with chemical immobilization procedures [1,2]. This physiological "dive response" is characterized by profound bradycardia, shunting of blood away from peripheral tissues, and periods of prolonged apnoea [3] that can be aggravated by the presence of immobilizing agents in the blood and tissues. This may result in relatively high concentrations of drug being transported to central organs, particularly the brain, which also affects the level of immobilization and recovery time [4]. The physiological status of an animal has also been shown to have a profound effect on sensitivity to immobilization and on the ability to metabolize chemicals [5]. Furthermore, the number of previous captures and immobilizations can increase recovery time [6]. Therefore, knowledge of the physiological (e.g., total body lipid) and anatomical characteristics (e.g., in some species the trachea is flat and has incomplete cartilaginous rings which may increase the risk of respiratory obstruction), methods of administration, and species-specific response to particular drugs are important for the effective, safe and optimal application of chemical immobilization in free-ranging wildlife species.

Intramuscular (IM) injection has been one of the most commonly used routes for administration of immobilizing agents in pinnipeds [see 1], and it is thought to be relatively safe and easy compared to other methods. Immobilization by intravenous (IV) injection has recently become more common with some species [6-8]. Although physical restraint is required prior to the administration of drugs using IV methods, smaller doses and better control of the intensity and duration of immobilization are generally achieved compared to IM injection methods. Pinnipeds that received the same drugs by IV and IM injection have been reported to have shorter induction and recovery times and less variable responses when IV methods were used [8-10].

Weddell seals (*Leptonychotes weddellii*) are deep-diving (> 500 m) predators that have been the subject of many studies requiring immobilization. Many of these studies have reported varying responses to immobilizing agents [11-15], and most have reported mortality rates ranging from 10 to 31 %, indicating that Weddell seals may be particularly sensitive. More recently, a safe method of gas anaesthesia (zero mortality) of Weddell seals has been reported by Kusagaya & Sato [16] and Bodley *et al*. [17] (n = 9, n = 11, respectively); however, this procedure is not always practical for field situations due to the cumbersome equipment required. Therefore, a reliable, safe and direct technique of immobilization is still required for this species.

A 1:1 mixture of tiletamine and zolazepam (available commercially as Telazol^®^, Fort Dodge, Castle Hill, Australia, or Zoletil^®^, Virbac, Peakhurst, Australia) has been characterized by rapid, smooth induction, good analgesia (unresponsive to painful stimuli), maintenance of pharyngeal and laryngeal reflexes, and a smooth recovery phase [18]. The tiletamine:zolazepam mixture has been used successfully to immobilize a range of domestic and wild mammals, including some pinniped species [6,7,14,19-25]. In its experimental stage (2:1 ratio mixture of tiletamine and zolazepam), Telazol was effective with Weddell seals [14]; however, some complications (i.e., apnoea leading to death) were encountered in a later study by Phelan & Green [12]. This may have been due to the method of administration rather than the drug itself (see Discussion). The 1:1 mixture of tiletamine:zolazepam potentially offers a safe and effective method for immobilizing Weddell seals in the field.

In this study we investigated the use of tiletamine:zolazepam (Telazol^®^) for Weddell seals. We compared performance between IV and IM injection, and examined the relationship between age, body condition (total body lipid, TBL) and stage of lactation on induction and recovery time. We hypothesized that variation in these parameters among individuals would influence drug sequestration and recovery time and that this may be more pronounced than at other stages in this species' life history due to the physiological pressures and energetic constraints of lactation [5]. In addition, we examined differences in recovery time due to the number of previous immobilizations. We hypothesized that recovery time would decrease for individuals that had been chemically immobilized previously, as has been found in other species [6].



## Results

**Figure 1:**
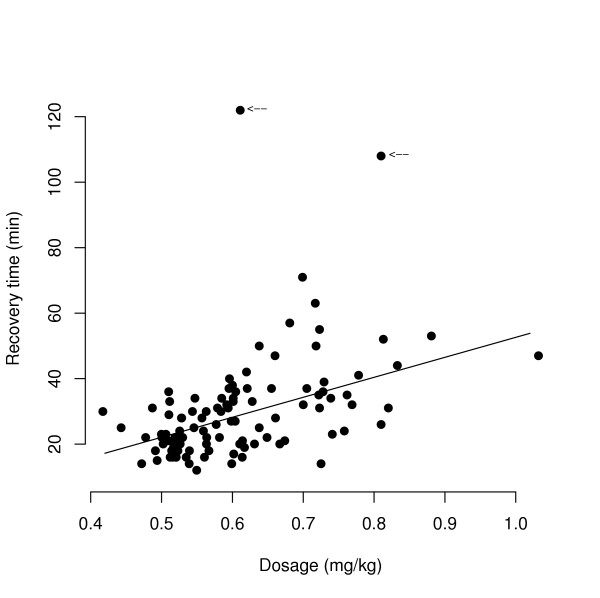
**Relationship between dosage and recovery time**. Linear relationship between dosage (mg/kg) of tiletamine and zolazepam (1:1 mixture) and recovery time (min) in lactating Weddell seals. Arrows denote two animals (outliers) with extended recovery times.

There was a strong linear relationship between dosage (mg/kg) and recovery time (GLM: information-theoretic evidence ratio, *ER *= 3.3 × 10^7^, per cent deviance explained, %DE = 21.0 %). Examination of two outliers revealed that there was nothing unusual about these individuals. Both were captured more than once and had average recovery times for the other captures. Exclusion of these outliers improved the relationship (*ER *= 8.2 × 10^7^, %DE = 31.0 %, Fig. 1). To control for the size of the seal and the level of immobilization, recovery times were weighted by the reciprocal of the dosage [6], referred to as 'weighted recovery time'

### Intravenous injection

#### Induction

**Figure 2:**
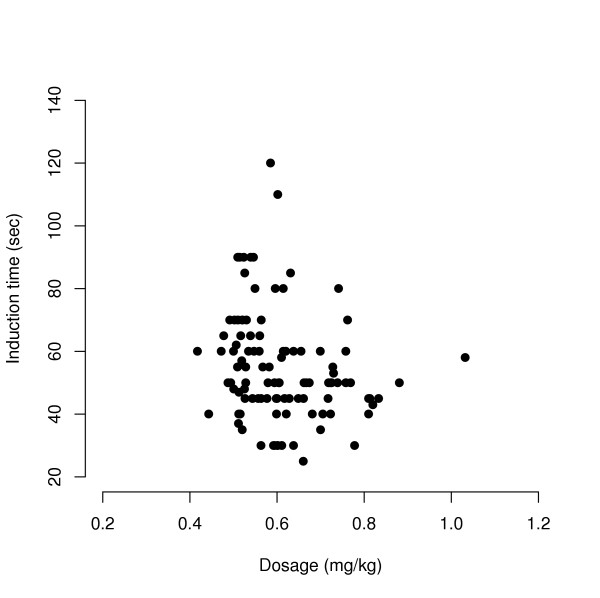
**Relationship between dosage and induction time**. Relationship between dosage (mg/kg) of tiletamine and zolazepam (1:1 mixture) and induction time (sec) in lactating Weddell seals.

**Figure 3:**
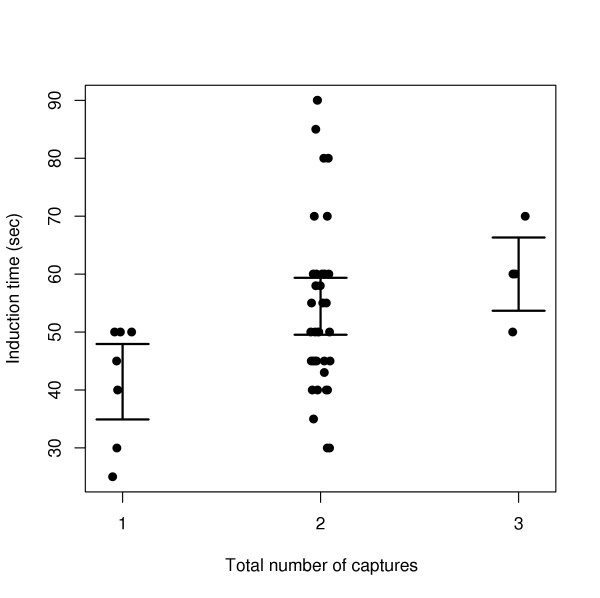
**Induction time relative to number of captures**. Average induction time (± 2 SE) of adult lactating Weddell seals immobilized with tiletamine and zolazepam (1:1 mixture). Induction time increased with the number of previous captures.

The mean dosage of tiletamine:zolazepam injected IV was 0.60 ± 0.01 mg/kg, with an average induction time of 54.8 ± 1.7 seconds (Fig. 2). Using information-theoretic weights of evidence [26] to examine the variation in induction time, there was no evidence that *TBL *or *age *affected induction time (Δ*w*+ ≤ 0 for both terms), but that *stage *(of lactation) had some effect (Δ*w*+ = 0.193), with induction time being longer at the end of lactation (beginning: 48.2 ± 2.9 seconds; end: 54.9 ± 2.3 seconds). We examined if there was an effect of the number of previous captures on induction time using a generalized linear mixed-effects model (GLMM). Here, c*apture *was the total number of captures experienced by that female and *induction *was the induction time measured for the last capture. The term *stage *was also included as a random effect to account for variation due to stage of lactation (the terms *capture *and *stage *were uncorrelated). The results revealed that *capture *explained 64.4 % of the variation in *induction *time (Fig. 3), indicating (via the evidence ratio) that this model was 7.47 times more likely to explain variation in induction time than the null model (i.e., a model with no effect of capture). Table 1 shows average induction times for each of the model predictors at average dose rates.

#### Recovery

Information-theoretic weights of evidence revealed that none of the terms considered explained the variation in weighted recovery times (Δ*w*+ ≤ 0 for all terms). The GLMM used to examine the influence of the number of previous captures on weighted recovery time revealed that *capture *only explained 21.0 % of the variance in weighted recovery time and the evidence ratio of this model to that of the null model was only 0.31, indicating no evidence of an effect of *capture *(see Table 1 for average recovery times for each of the model predictors).

### Intramuscular injection

Only 4 females were injected intramuscularly with the tiletamine:zolazepam mixture. Three of these were post-partum captures and one was an end-lactation capture. Average dosage was 0.9 ± 0.6 mg/kg, with an average induction time of 15.3 ± 1.5 minutes. One female was immobilized (IM) both post-parturition and at the end of lactation. No problems were associated with her first immobilization procedure. During her second capture at the end of lactation, induction was fast (4 minutes), indicating possible accidental intravenous injection. Regular shallow breathing was maintained through most of the procedure. However, after approximately 60 minutes she experienced a prolonged apnoeic event, was unresponsive to resuscitation procedures and subsequently died.

## Discussion

Telazol administered intravenously was an effective drug for the immobilization of Weddell seals. However, intramuscular administration was less successful with a longer induction and recovery times and a 25 % (one animal in four) mortality rate. The IM route of injection has been previously favoured because physical restraint is often not required so administration is easy and safe for personnel. Nonetheless, the IM route of administration has some disadvantages. Accidental injection into the blubber (which can be > 50 mm in adult Weddell seals) can lead to variable induction and recovery times. Furthermore, how quickly the drug is absorbed into the bloodstream depends, in part, on the blood supply to the muscle. Blood supply increases during physical activity, which could account for the deaths reported by Phelan & Green [12] because they physically handled and restrained females before injection. In this study, females were only immobilized IM when IV injection was impossible (due to this species' tendency to 'roll' when restrained), so individuals were physically handled and potentially agitated similar to those in the Phelan & Green study. The single death in this study may have resulted from the accidental injection of a larger amount of drug into a vein. Other studies administering the tiletamine:zolazepam mixture IM have also shown variable results, especially with higher doses (Table 2), although these may have also resulted from accidental injection IV. Taken together, these observations suggest that the tiletamine:zolazepam combination may have a narrow margin of safety in some seal species when administered IM, and that IM administration increases the risks associated with immobilization. By contrast, IV administration may be an acceptable alternative.

Weddell seals appear to be more sensitive, to some extent, than other species to drug type [11,13-15] and method of administration. Some drugs (e.g., phencyclidine HCl and succinylcholine chloride) that have lead to fatalities in Weddell seals have also had variable and lethal results with other similar-sized phocids [1]. Ketamine HCl, a rapid acting dissociative with a similar molecular structure to phencyclidine HCl, has also been lethal to Weddell seals, but not to other species at similar dosages [1]. In general, Weddell seals appear to respond to tiletamine and zolazepam in a similar way to southern elephant seals, but we can only speculate as to why differences might exist for other drug types. Weddell seals live in an extreme environment year round and their energetic adaptations might influence their sensitivity and response.

In this study, chemical immobilization with tiletamine and zolazepam using the IV method was successful in all cases. There was a small effect of stage of lactation on induction time, with an increase of about six seconds (12.2 %) at the end of lactation. The number of previous captures appeared to increase induction time (although sample size was admittedly low for animals immobilized more than twice), suggesting a decrease in sensitivity to the chemical when first introduced into the bloodstream, even up to 2–3 weeks later. On the other hand, there were no detectable effects of age, condition, stage of lactation or number of previous captures on weighted recovery time. The animals immobilized were under varying degrees of physiological pressures associated with the negative energy balance of lactation [5]. It seems reasonable that if physiological state was to affect weighted recovery time it would be most evident in these individuals.

Previous studies on southern elephant seals have shown an effect of age and condition on recovery time [5,6]. However, Woods *et al*. [5] found no significant difference in recovery time between post-parturition and end-of-lactation females, although they did find that pre-moult seals (i.e., in better condition) had shorter recovery times. The differences between individuals found in the Field *et al*. [6] study were based on measurements of condition and recovery times at three different haul-out periods separated by months as opposed to weeks in our study. Therefore, our results in combination with the findings of Woods *et al*. [5] suggest that recovery time does not differ between physiologically stressed animals within the same state (e.g., lactation), although differences resulting from changes in physiological status at other times (e.g., non-moult to moult) may affect recovery times enough to be measurable in field studies.

Although we did not find a difference in weighted recovery time and number of captures, our sample size was low. With a larger sample size and more repeated captures (up to 5), Field *et al*. [6] found a positive relationship between weighted recovery time for southern elephant seals and the number of times an individual had been immobilized previously. However, this relationship was not as apparent up to 3 captures (as in this study), so it is possible that we did not have the statistical power to detect a relationship. Consequently, we suggest careful monitoring of immobilization recovery times during longitudinal studies (≥ 3 captures) on Weddell seals to examine this potential relationship further.

## Conclusion

The 1:1 mixture of tiletamine and zolazepam appears to be a suitable and safe drug for intravenous immobilization of Weddell seals. It appears that variability in recovery rates generally increases with higher doses (Fig. 1) likely due to individual differences in the rates of metabolism and elimination of tiletamine and zolazepam [21]. Taking these differences into consideration, we recommend a mass-specific dose of 0.50 – 0.65 mg/kg which gives an average recovery time of 26 minutes that should be suitable for most procedures requiring immobilization (e.g., deployment of dataloggers, tissue sampling, injection of isotopic compounds to examine body composition, etc.). This corresponds to the dosage recommended for southern elephant seals by McMahon *et al*. [7]. Although immobilization techniques will vary for species and situations, this suggests that a mass-specific dose of 0.50 mg/kg may be a good starting point for other age classes and other phocid seals.

## Methods

### Field procedures

A total of 110 chemical immobilization procedures using Telazol (1:1 mixture of tiletamine and zolazepam) were done on adult female Weddell seals as part of a study on maternal energy expenditure and lactation energetics. One hundred and six of these were by IV injection and 4 were by IM injection. Some individuals were immobilized more than once during the course of their lactation period (5–6 weeks), but no individuals were immobilized more than three times. Females were caught on the sea ice at Erebus Bay, Antarctica (77° 51' S, 166° 45' E) during the breeding season (October to December) of 2002 and 2003. Individuals were identified by flipper tags attached in previous years as part of a long-term tagging study [27], and ages ranged from 6 to 22 years old.

Females were easily approached on the ice and pups were relocated several metres away to avoid potential injury. Subsequently, a canvas bag was placed over the female's head [7], after which the majority of individuals remained in a prone position without struggle. The few that were slightly agitated would commence a 'rolling' behaviour and could not be restrained effectively on the ice. However, this behaviour typically ceased within 2–3 minutes. Females were then injected with Telazol intravenously via the extra-dural vein in the lumbar region [7] using a 5 ml syringe and 15 cm (6") 18G spinal needle, or intramuscularly in the rear flank with a 10 ml syringe and 9 cm (3.5") 18G needle. We attempted to give dosages of 0.5 mg/kg [7] and 0.75 mg/kg [14] IV and IM, respectively. Dosages at the first capture were calculated using an estimate of female body weight based on researcher's previous experience working with phocids. For additional captures, dosages were calculated by estimating mass loss rates through lactation. Drug induction and recovery times were recorded and the respiratory rate and volume of air moving (as estimated by listening to breathing sounds) were monitored throughout procedures. Induction time (seconds) was defined as the time from injection until the animal did not respond to a tap on the nose [7]. The recovery time (minutes) was defined as the time from immobilization until the seal responded to a tap on the nose by moving and raising its head and maintaining its head in a raised position for ~10 seconds [28]. This was repeated several times to ensure complete recovery. An endotracheal tube, oxygen, doxapram hydrochloride (Dopram^®^, Wyeth, Baulkham Hills, Australia) and flumazenil (Anexate^®^, Roche, Castle Hill, Australia) were available in the event of respiratory arrest.

After immobilization, females were weighed to the nearest 1 kg using electronic scales and standard body length and six girth measurements [G1-G6, 6] were recorded. The precise dosages of tiletamine:zolazepam were calculated for each female based on measured weights. Body composition (i.e., proportion of lipid and lean tissues) was measured using an isotopically labelled water technique. A 10 ml blood sample was collected to measure background isotope levels followed by the IV injection of a pre-weighed dose (to the nearest 0.1 mg) of 222 MBq tritiated water (HTO) into the extradural vein. The syringe was flushed with blood twice to ensure complete isotope delivery. A second blood sample (10 ml) was taken approximately 150 minutes after initial injection for the calculation of dilution space and body composition. Houser & Costa [29] found that HTO equilibration occurs within 90 minutes of an intravenous injection of northern elephant seal (*Mirounga angustirostris*) pups. Equilibration occurs in southern elephant seal (*M. leonina*) pups within 120 minutes of administration (IV; K.E. Wheatley, unpublished data). Therefore, we considered 150 minutes to be sufficient time before collecting a second blood sample. All samples were stored at -20°C until analysis.

### Laboratory analysis

Plasma samples were analysed for HTO activity using liquid scintillation spectrometry. Plasma samples (100 μl) were distilled in triplicate using the method of Ortiz *et al*. [30]. For each vial of water recovered, 4 ml of EcoLite scintillate (ICN, Costa Mesa, USA) was added and HTO activity was counted for 15 minutes using a Beckman LS6500 scintillation counter. Correction for quenching was made by automatic external standardization. Calculations of body composition were done as described by Reilly & Fedak [31].

### Data analysis and calculations

We did not obtain body composition data for 37 captures, but for 11 of these animals we obtained composition data for captures before and after the capture in question. Body composition for this intermediate capture was estimated by interpolation, assuming the change in composition was linearly proportional to a change in mass.

A set of generalized linear models (GLM) and penalized quasi-likelihood [PQL, 32] generalized linear mixed-effects models (GLMM) were constructed to examine the relationships between recovery and induction time and the various state variables. GLMs extend the standard regression model by (1) distributing the response *y *about its expected value *μ *according to a distribution F (e.g., normal, gamma, binomial, etc.), and (2) entering the predictors *x*_1_, *x*_2_,., *x*_*m *_into the model through the linear predictor *η*, which is related to the expected response *μ *by a monotonic link function *η*_*i *_= *η*(*μ*_*i*_) [33]. GLMMs are linear models that include both fixed and random effects, where random effects are those associated with individual experimental units drawn at random from a population [e.g., individuals as in this study, 34]. GLMMs offer the advantage of partitioning variances due to the effects under investigation (fixed) and those that do not contribute to the hypotheses being tested (random).

Model comparison used Kullback-Leibler information to assign relative strength of evidence (Akaike's Information Criterion corrected for small samples [AICc, 26, 35]) to each model in the set [26]. To compare a more complex model *a *to a simpler model *b*, we employed the information-theoretic evidence ratio (*ER *= AIC_*c *_weight of model *a *÷ AIC_*c *_weight of model *b*) to quantify the relative support of *a *versus *b*, and used the per cent deviance explained (%DE) to determine structural goodness-of-fit of model *a *(test for model adequacy). Higher *ER *values indicate higher likelihoods of the tested model relative to model *b *(e.g., the null model).

The weights of evidence (*w+*_*i*_) for each predictor were calculated by summing the model AIC_*c *_weights (*w*_*i*_) over all models in which each term appeared. However, the *w*+_*i *_values are relative, not absolute because they will be > 0 even if the predictor has no contextual explanatory importance [26]. Therefore, a baseline for comparing relative *w*+_*i *_across predictors is required to ascertain which predictors are relevant. We randomized the data for each predictor separately within the dataset, re-calculated *w*+_*i*_, and repeated this procedure 100 times for each predictor. The median of this new randomized *w*+_*i *_distribution for each predictor was taken as the baseline (null) value (*w*+_0_). For each term the relative weight of evidence (Δ*w*+) was obtained by subtracting *w*+_0 _from *w*+_*i*_. Predictors with Δ*w*+ of zero or less have essentially no explanatory power [26].

To account for repeated captures (measurements), a series of GLMMs were constructed to examine relationships between induction and weighted recovery times and the age, total body lipid, stage of lactation and total number of captures. Examination of the residuals for the GLMMs determined that the gamma error distribution family and an identity link function were the most appropriate for each analysis. All statistical analyses were done using the R Package [Ver. 2.0.1, 36]. Values are presented as mean ± one standard error (SE) unless otherwise stated.

## Authors' contributions

KEW collected the data, did the laboratory analysis and drafted the manuscript. CJAB provided valuable assistance with the statistical analysis and critically revised the manuscript. RGH and LSD participated in collection of data and critically revised the manuscript. MAH critically revised the manuscript and provided important intellectual content. All authors read and approved the final manuscript.

## Figures and Tables

**Table 1:** Summary of induction and recovery times at average dose rates for different physiological conditions tested

		Dosage (mg/kg)		Induction (sec)		Recovery (min)	
	Range	Mean	SE	Mean	SE	Mean	SE

Capture	1	0.60	0.01	49.5	2.98	32.2	1.61
	2	0.52	0.00	64.4	2.88	20.4	0.97
	3	0.69	0.02	54.9	2.24	30.6	2.27
Age (yr)	6 – 9	0.62	0.02	48.4	1.95	29.1	1.91
	10 – 14	0.60	0.02	57.5	2.84	26.9	2.07
	15 +	0.57	0.02	61.3	6.07	29.7	3.20
% TBL	21 – 33	0.65	0.02	52.7	2.74	27.8	2.51
	34 – 40	0.62	0.02	57.9	3.20	27.6	1.78
	41 +	0.62	0.02	48.6	3.39	35.2	2.40

**Table 2:** Summary of dosages of tiletamine:zolazepam (1:1) used for chemically immobilizing phocid seals

**Species**	***n***	**Dosage (mg/kg)**	**Route**	**Mortality %**	**Reference**
*Halichoerus grypus *Grey seal	44	1.0	IM dart	0	[24]
*Phoca vitulina *Harbour seal	7	0.75 – 2.0	IM	0	[14]
*Hydrurga leptonyx *Leopard seal	1	2.0	IM	100	[37]
*Leptonychotes weddellii *Weddell seal	30	0.67	IM	10	[12]
	4	0.86	IM	25	this study
	106	0.60	IV	0	this study
*Mirounga leonina *Southern elephant seal	90	1.0	IM dart	0	[24]
	5	2.0	IM	40	[37]
	4	0.6 – 1.7	IM	0	[19]
	597	0.46	IV	0	[7]
	1033	0.53	IV	0	[6]

